# Life-space, frailty, and health-related quality of life

**DOI:** 10.1186/s12877-022-03355-2

**Published:** 2022-08-06

**Authors:** Petronella Chitalu, Alex Tsui, Samuel D. Searle, Daniel Davis

**Affiliations:** 1 MRC Unit for Lifelong Health and Ageing at UCL, 1-19 Torrington Place, London, WC1E 7HB UK; 2 Division of Geriatric Medicine, Dalhousie University, Nova Scotia, Canada

**Keywords:** Life-space, Frailty, Quality of life, Epidemiology

## Abstract

**Introduction:**

Life-space and frailty are closely linked to health-related quality of life and understanding their inter-relationship could indicate potential intervention targets for improving quality of life. We set out to examine the relationship between frailty and life-space and their relative impact on quality of life measures.

**Methods:**

Using cross-sectional data from a population-representative cohort of people aged ≥ 70 years, we assessed quality of life with the EuroQol Health Index tool (5-levels) (EQ-5D-5L). We also undertook a life-space assessment and derived a frailty index. Linear regression models estimated EQ-5D-5L scores (dependent variable) using life-space assessment, frailty index and interactions between them. All models were adjusted by age, sex, lifestyle, and social care factors.

**Results:**

A higher EQ-5D Index was associated with higher life-space (0.02 per life-space assessment score, 95%CI: 0.01 to 0.03, *p* < 0.01) and decreasing frailty (-0.1 per SD, 95%CI: -0.1 to -0.1, *p* < 0.01). There was evidence of an interaction between life-space and frailty, where the steepest gradient for life-space and EQ-5D was in those with the highest frailty (interaction term = 0.02 per SD of frailty, 95%CI: 0.01 to 0.03, *p* < 0.01).

**Conclusion:**

Individuals with the highest frailty were twice as likely to have higher quality of life in association with a larger life-space. Interventions designed to improve quality of life in frail older people could focus on increasing a person’s life-space.

**Supplementary Information:**

The online version contains supplementary material available at 10.1186/s12877-022-03355-2.

## Introduction

Maintaining health-related quality of life (HRQoL) into older age is a key ambition for multidisciplinary healthcare teams [1, 2]. Quality of life has several dimensions, covering the physical, psychological, and social aspects of an individual’s well-being and function [3]. Other multidimensional quantities relevant to older people include frailty and life-space [4, 5]. Frailty results from cumulative decline across multiple physiological systems. Life-space assessments integrate several aspects of functional mobility. Each measure is closely linked, and understanding their inter-relationship could indicate potential intervention targets for improving quality of life [2, 6].

Life-space is a rich and informative assessment of mobility with good construct validity, yet it is not yet well-established as a clinical tool [7]. Higher scores reflect greater degrees of function, which can be multiplied to give a single measure [8]. An individual’s total life-space is dynamic and may change as a result of acute (e.g., after stroke or surgery) and chronic (e.g., dementia or osteoarthritis) health conditions [9, 10]. Smaller life-space is associated with a lower quality of life [11], though how changes in life-space impact quality of life across the spectrum of frailty has not been described [12]. Frailty is an important contextual factor given that frail and pre-frail individuals have greater life-space decline than non-frail individuals [13].

We set out to examine the relationship between life-space, frailty, and their relative impact on overall quality of life in a population-representative cohort. We investigated variables related to quality of life and life-space, hypothesising that increasing life-space would be associated with improving quality of life to varying degrees between frail and robust older adults.

## Methods

We used data collected between 2018 and 2019 from the Delirium and Population Health Informatics Cohort (DELPHIC). Details have already been published [14–16]. In brief, DELPHIC is a population-based prospective study of residents aged ≥ 70 years in the London Borough of Camden. The sample was mainly enrolled from primary care lists and is representative of the borough in terms of age distribution and income deprivation indices. Here, we used data from the first 1,510 individuals recruited. This is a cross-sectional analysis of the baseline assessment.

### Outcome

Quality of life was defined by the EuroQol Health Index tool (5-levels) (EQ-5D-5L) [17], which includes a visual analogue scale (VAS) summarising a self-rating for quality of life from 0 to 100 (100 = ‘best health’). EQ-5D-5L also has domains on mobility, self-care, usual activities, pain/discomfort, and anxiety/depression. Using empirical value sets for an English population, a score in each domain generates an overall EQ-5D index where 0 is equivalent to dead (negative values mean ‘worse than dead’) and 1 refers to ‘full health’ (values above 1 indicate even higher health utility).

### Exposure

The life-space assessment is a self-reported measure of an individual’s independent mobility [18]. It relates to the dimensions of geographical space in which a person’s life takes place. Life-space has three components: distance travelled (5 levels, from bedroom only to beyond the neighbourhood); frequency of travel (4 levels, from daily to < 1/week); need for assistance (3 levels, none / with equipment / with personal assistance). Responses refer to the previous four weeks’ activity. Multiplying these scores indicates an individual’s functional mobility (range 0 to 120). Higher scores indicate greater mobility.

### Covariates

We included health, social and lifestyle factors, such as frailty, frequency of contact with next of kin, and receiving a care package. We selected these based on previously reported associations with quality of life [19, 20] and life-space [7]. These items were self-reported and collected by an interviewer who could corroborate data through real-time access to health and social care records. Distance from next of kin was documented in miles and living alone was recorded as ‘yes’ or ‘no’. Contact with a next of kin was categorised as in-person or by phone, recorded as daily, weekly, monthly, yearly, or less. Monthly or less frequent contact with a next of kin was used to define isolation. Care package receipt was recorded by frequency (from hired cleaners only up to 24-h care). For health behaviours, cigarette smoking status was asked directly and classified as: current smoker, ex-smoker and non-smoker, and alcohol consumption was recorded as daily, weekly, monthly or less frequently. Frailty was quantified using a Frailty Index, representing the proportion of accumulated health deficits, including comorbidities. The Frailty Index ranges from 0 to 1, with higher values indicating more frailty. We derived it using 35 items from the baseline assessment, covering general health, comorbidities, medications, health behaviours, hearing, vision, dental health, continence, falls, depression, personal and instrumental activities of daily living, and calculated according to standard procedures [21] (Supplementary table 1). Tertiles of frailty were: low frailty up to 0.08; medium frailty 0.09 to 0.24; high frailty 0.25 or greater [22]. Socioeconomic position (SEP) was operationalised using highest educational attainment (primary/secondary/tertiary), the Office for National Statistics occupational skill classification (4 levels) and Index of Multiple Deprivation (IMD) score, an ecological measure where higher scores indicate neighbourhood disadvantage [23, 24].

### Statistical analysis

We used a series of linear regression models to estimate the associations between our continuous outcomes (EQ-5D index, visual analogue scale) and covariates. We used median imputation for any data missing within the life-space assessment dimensions and multiple imputation (20 imputations) for other missing covariate data. We assessed interactions between life-space and frailty index scores by creating a parameter multiplying the two continuous measures, testing the model with and without this interaction term. To improve comparability and ease of interpretation, we transformed these into standardised z-scores (score-mean)/standard deviation). We used Stata version 16.1 for all analyses (StataCorp LLC, College Station, Texas).

## Results

The mean age of the full sample was 78 (SD 6.2), and 41% were men, and most individuals were educated to degree, or postgraduate level and had high-skill occupations (Table 1). Outcome scores were missing in 24% of participants, with 5% missing both EQ-5D Index and the Visual Analogue Scale score. Missing outcome scores were more likely in people with higher frailty (FI 0.19 versus 0.15) and with more multimorbidity (1.4 versus 1.6 conditions) (Table 1). The remainder of the analyses were on participants with available EQ-5D Index and Visual Analogue Scale data (*n* = 1152). Individuals in the middle tertile of EQ-5D Index (between 0.7 and 0.8) had a Visual Analogue Scale score of 79/100. Participants reported an average life-space of 66/120, broadly equivalent to someone who is able to leave their neighbourhood several times a week with the assistance of equipment. The mean frailty index was 0.15 (Table 1). Table 2 describes typical clinical presentations of different levels of life-space by degree of frailty.

**Table 1:** Descriptive characteristics of the sample (*n* = 1510, missing EQ-5D-5L Index = 358 (24%))

				EQ-5D-5L Index			
	Total sample (*n* = 1510)	Missing Quality of life (Both measures)	*P*	< 0.70 (*n* = 324)	0.70 to 0.80 (*n* = 428)	> 0.80 (*n* = 400)	*P*
	n or mean	n or mean		n or mean	n or mean	n or mean	
Men	625 (41%)	27 (2%)	0.305	133 (43%)	178 (43%)	172 (44%)	0.875
EQ-Visual Analogue Scale (SD)	78.6 (15.6)			78.6 (16.4)	79.1 (15.5)	78.7 (15.4)	0.928
Life-space (score, SD)	65.7 (17.5)	82 (53.7)	0.186	64.7 (18.5)	66.3 (17.4)	65.5 (17.6)	0.585
Frailty index (SD)	0.15 (0.13)	0.19 (0.15)	0.005	0.15 (0.12)	0.14 (0.12)	0.16 (0.14)	0.447
Age (years, SD)	78 (6.2)	78 (6.0)	0.488	78 (6.4)	78 (5.9)	78 (6.2)	0.774
Multimorbidity (count, SD)	1.4 (1.2)	1.6 (1.3)	0.039	1.3 (1.1)	1.4 (1.3)	1.4 (1.1)	0.488
IMD (deprivation score, SD)	16.6 (9.1)	17.9 (8.9)	0.226	15.8 (8.5)	17.1 (9.6)	17.0 (9.1)	0.125
Education							
Up to primary	213 (14%)	12 (1%)	0.44	50 (16%)	52 (12%)	58 (15%)	0.679
Up to secondary	313 (21%)	21 (1%)		62 (19%)	89 (21%)	83 (21%)	
Degree level	968 (64%)	47 (3%)		210 (65%)	285 (67%)	251 (64%)	
Occupational skill level							
Level 1	81 (5%)	6 (0.4%)	0.068	21 (7%)	21 (5%)	22 (6%)	0.446
Level 2	237 (16%)	13 (1%)		46 (14%)	59 (14%)	68 (17%)	
Level 3	246 (16%)	20 (1%)		41 (13%)	70 (16%)	67 (17%)	
Level 4	935 (62%)	39 (3%)		215 (67%)	276 (65%)	242 (61%)	
NOK contact (in person)							
Daily or weekly	1099 (73%)	63 (4%)	0.411	237 (75%)	325 (76%)	286 (72%)	0.485
Monthly	211 (14%)	8 (1%)		44 (14%)	57 (14%)	61 (15%)	
Yearly or less	167 (11%)	7 (0.5%)		35 (11%)	37 (9%)	48 (12%)	
NOK contact (by phone)							
Daily or weekly	1026 (68%)	53 (5%)	0.231	222 (88%)	299 (91%)	267 (86%)	0.372
Monthly	97 (6%)	3 (0.3%)		21 (8%)	22 (7%)	34 (11%)	
Yearly or less	40 (3%)	0 (0%)		9 (4%)	9 (3%)	11 (4%)	
Care package							
None	1413 (94%)	73 (5%)	0.632	303 (94%)	405 (95%)	370 (93%)	0.706
Weekly	26 (2%)	2 (0.1%)		4 (1%)	7 (2%)	8 (2%)	
Daily or more	68 (5%)	5 (0.3%)		16 (5%)	16 (4%)	22 (6%)	
Smoking status							
Never	640 (42%)	30 (2%)	0.447	144 (45%)	179 (42%)	170 (43%)	0.907
Ex-smoker	774 (51%)	43 (3%)		160 (50%)	226 (53%)	203 (51%)	
Current	91 (6%)	7 (0.5%)		19 (6%)	23 (5%)	25 (6%)	
Alcohol intake							
Daily	534 (35%)	30 (2%)	0.12	110 (39%)	140 (41%)	149 (44%)	0.754
Weekly	458 (30%)	16 (1%)		112 (40%)	134 (39%)	120 (35%)	
Monthly or less	267 (18%)	18 (1%)		60 (21%)	71 (21%)	71 (21%)	
Distance from NOK (miles, SD)	201.9 (1299.2)	299 (1242.9)	0.507	120.2 (595.3)	151.1 (1088.2)	207.1 (1074.2)	0.478

Items all assessed by interview or self-reported

*IMD* Index of Multiple Deprivation, *NOK* Next of kin

**Table 2:** Life-space scores and profiles according to frailty

	Low	Medium	High
Life-space: mean (SD)	74.3 (11.5)	66.9 (14.0)	41.4 (20.0)
Description of typical individual	Independently travels outside city on a weekly basis	Mobilises outdoors independently but rarely beyond their neighbourhood	Leaves house daily or able to but needs rollator frame; leaves neighbourhood rarely and would need personal assistance to do so

**Fig. 1:**
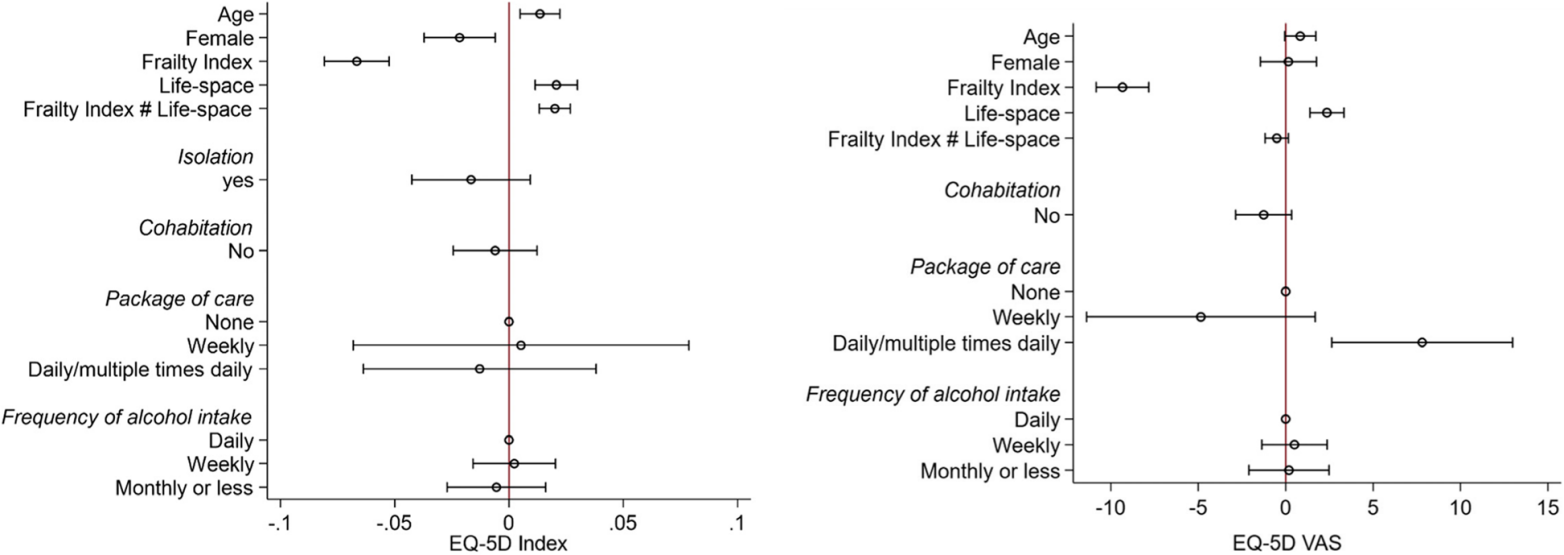
Variables associated with HRQoL

A higher EQ-5D Index was associated with older age (0.01 per year, 95%CI: 0.005 to 0.02, *p* < 0.01), higher life-space (0.02 per life-space assessment score, 95%CI: 0.01 to 0.03, *p* < 0.01) and decreasing frailty (-0.1 per SD, 95%CI: -0.1 to -0.1, *p* < 0.01) (Table 3, Fig. 1). Women had a lower EQ-5D Index (-0.02, 95%CI: -0.04 to -0.01, *p* < 0.01). Similar patterns were evident for the Visual Analogue Scale scores. Neither self-reported isolation, frequency of contact, nor distance from next of kin were associated with quality of life.

**Table 3:** Variables associated with quality of life (*n* = 1,152)

	EQ-5D Index								Visual Analogue Scale							
	Unadjusted				Multivariate Linear Regression of 943 individuals				Unadjusted				Multivariate Linear Regression of 1,152 individuals			
	Coef	[95% Conf. Interval]		*P*	Coef	[95% Conf. Interval]		*P*	Coef	[95% Conf. Interval]		*P*	Coef	[95% Conf. Interval]		*P*
Age	-0.03	-0.04	-0.02	< 0.001	0.01	0.005	0.02	0.002	-3.6	-4.5	-2.7	< 0.001	0.8	-0.1	1.7	0.068
Women (cf.) Men	-0.03	-0.04	-0.01	0.003	-0.02	-0.04	-0.01	0.007	0.1	-1.7	2.0	0.873	0.2	-1.5	1.8	0.851
Frailty index	-0.1	-0.1	-0.1	< 0.001	-0.1	-0.1	-0.1	< 0.001	-9.5	-10.4	-8.6	< 0.001	-9.3	-10.8	-7.8	< 0.001
Life-space	0.1	0.1	0.1	< 0.001	0.02	0.01	0.03	< 0.001	6.0	5.2	6.7	< 0.001	2.4	1.4	3.3	< 0.001
Frailty Index # Life-space					0.02	0.01	0.03	< 0.001					-0.5	-1.2	0.2	0.134
NOK contact (in person)																
Daily/weekly	[ref]			0.047					[ref]			0.174				
Monthly	-0.01	-0.03	0.02						-0.9	-3.5	1.7					
Yearly or less	-0.04	-0.1	-0.01						-2.6	-5.4	0.2					
NOK contact (by phone)																
Daily/weekly	[ref]			0.051					[ref]			0.219				
Monthly	-0.04	-0.1	-0.005						0.2	-3.3	3.8					
Yearly or less	-0.03	-0.1	0.02						-4.6	-9.8	0.6					
Isolation	-0.04	-0.1	-0.01	0.010	-0.02	-0.04	0.01	0.210	-1.8	-4.8	1.3	0.252				
Lives alone	-0.03	-0.05	-0.01	0.001	-0.01	-0.02	0.01	0.518	-3.0	-4.8	-1.2	0.001	-1.3	-2.9	0.3	0.123
Distance from NOK (miles)	-0.001	-0.01	0.01	0.809					0.001	-0.9	0.9	0.999				
Care package																
None	[ref]			< 0.001				0.853	[ref]			< 0.001				0.001
Weekly	-0.2	-0.2	-0.1		0.01	-0.1	0.1		-23.8	-30.8	-16.8		-4.9	-11.4	1.7	
Daily/multiple times daily	-0.3	-0.4	-0.3		-0.01	-0.1	0.04		-15.6	-20.2	-11.1		7.8	2.6	13.0	
Smoking status																
Never	[ref]			0.533					[ref]			0.168				
Ex-smoker	-0.01	-0.02	0.01						-0.8	-2.7	1.0					
Current	-0.02	-0.1	0.02						-3.7	-7.6	0.3					
Alcohol intake																
Daily	[ref]			< 0.001				0.769	[ref]			< 0.001				0.886
Weekly	-0.004	-0.03	0.02		0.002	-0.02	0.02		0.1	-2.1	2.3		0.5	-1.4	2.4	
Monthly or less	-0.06	-0.08	-0.04		-0.01	-0.03	0.02		-4.7	-7.3	-2.2		0.2	-2.1	2.5	

*NOK* Next of kin

Lower life-space was associated with older age (-2.5 per year (95%CI: -3.2 to -1.7, *p* < 0.01) and frailty (-6.6 per SD, 95%CI: -7.4 to -5.7, *p* < 0.01) (Supplementary Table 2, Supplementary Fig. 1). Women had lower life-space (-2.0, 95%CI: -3.4 to -0.5, *p* < 0.01). There was a graded association between educational attainment and life-space, such that those with no qualifications had the lowest life-space (-3.5, 95%CI: -6.3 to -0.7, *p* = 0.03).

**Fig. 2:**
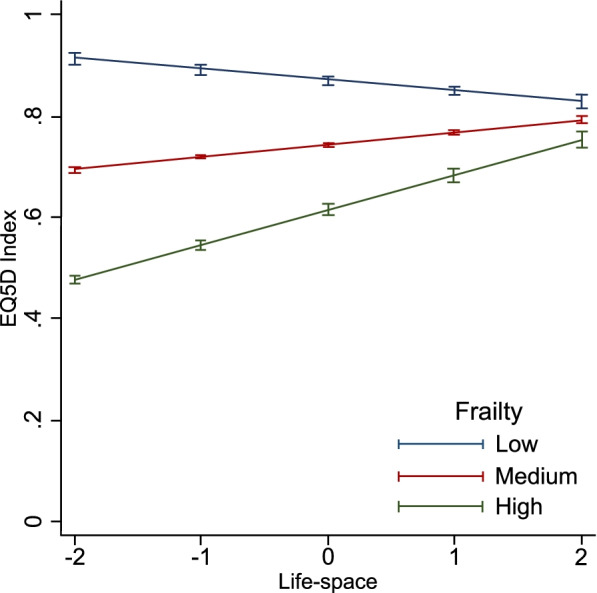
Interaction between life-space and frailty and their association with EQ-5D

We found evidence of an interaction between life-space and frailty and associated EQ-5D (Table 3, Fig. 2). The steepest gradient for life-space and EQ-5D was in those with the highest frailty (interaction term = 0.02 per SD of frailty, 95%CI: 0.01 to 0.03, *p* < 0.01). This coefficient was the same as the association between life-space and EQ-5D, which translates to a doubling of the effect size for each SD of increasing frailty. The highest effect was in those with a high level of frailty compared to those with a low and medium level of frailty. Improving indoor and outdoor mobility (and frequency of being outdoors) in the middle range of life-space (-1SD to + 1SD), would be associated with a 0.1 point (one tertile) improvement in quality of life (Table 2). This difference was comparable to an individual 10 years younger. Conversely, additional gains in life-space were not associated with better quality of life for those with low frailty, indeed these were slightly lower. The estimated coefficients for these relationships between life-space and quality of life was as follows: highest frailty (0.06, 95%CI: 0.03 to 0.08, *p* < 0.01); medium frailty (0.02, 96%CI: -0.002 to 0.04, *p* < 0.01), compared to low frailty (Supplementary Table 3).

## Discussion

Health-related quality of life appears to depend on both life-space and frailty, even after adjustment for domestic contact, isolation and need for social care. While life-space and frailty are closely related, their associations with quality of life vary depending on the underlying level of frailty. Life-space and quality of life have a stronger association in those with high frailty. Taken together, these findings suggest that targeted improvements in life-space mobility may be most beneficial for quality of life in older adults with high frailty compared to those with a low and medium level of frailty.

Our results are consistent with studies separately demonstrating the two associations between frailty and quality of life [25, 26] and life-space and quality of life [27]. However, showing how changes in life-space could impact quality of life across the spectrum of frailty is novel. Our findings emphasise the importance of understanding the determinants of life-space and how interventions in this domain could improve quality of life. The nature of this interaction would suggest that interventions to improve life-space could have the largest impact in those already living with frailty. The degree to which life-space could be modified has not been extensively studied. In a study of post-acute patients recently discharged, inpatient rehabilitation did not appear to improve life-space [28]. Similarly, although a resistance and balance training programme decreased falls risk, it did not increase life-space in care home residents [29]. After knee arthroplasty, patients who were less frail (by selection) and receiving an extended walking intervention showed improved life-space [30]. However, a multidisciplinary team community rehabilitation intervention demonstrated greater life-space in frail patients, even after 12 months [31]. In this respect, it might be expected that such interventions could also lead to improvements in quality of life. Overall, we interpret the interaction between life-space and frailty as identifying a subpopulation of individuals, those with most frailty, for whom mobility-related goals might make the biggest difference to their quality of life.

The cross-sectional nature of the data limits our findings, so we cannot establish any temporal relationships. We had missing quality of life data for one quarter of the sample, with the likely effect that this under-estimated the associations with life-space and frailty. Simultaneously comparing data on life-space, frailty and quality of life required us to standardise and transform the independent variables. Though we could establish overall relationships, it is difficult to link the estimated models directly to absolute levels of frailty. As with other observational studies, our results are subject to residual confounding. It is also not possible to generalise our findings outside the sample’s predominantly white, well-educated urban setting. Nonetheless, population cohorts have the advantage of offering data on the full range of life-space and frailty states.

In a population-representative cohort of older people, we demonstrate that life-space has the strongest relationship with quality of life in frail older adults. Frail individuals were twice as likely to have higher quality of life in association with a larger life-space. Interventions designed to improve quality of life in frail older adults could focus on increasing a person’s life-space.

## Supplementary Information

**Additional file 1: Supplementary Table 1.** Frailty Index creation.

**Additional file 2: Supplementary Table 2.** Variables associated with life-space (*n*=1,510).

**Additional file 3: Supplementary Table 3.** Variables associated with HRQoL (with frailty in levels)

**Additional file 4: Supplementary Figure 1.** Variables associated with life-space.

## Data Availability

The datasets generated and/or analysed during the current study are available in the Dementias Platform UK (DPUK) repository, https://portal.dementiasplatform.uk/CohortDirectory/Item?fingerPrintID=DELPHIC.
