# Supplementary material for: Life-space, frailty, and health-related quality of life
Source: BMC Geriatr. 2022 Aug 6;22:646. doi: 10.1186/s12877-022-03355-2 (PMC9356461; doi:10.1186/s12877-022-03355-2)
Supplement: Supplementary file 1 — Additional file 1: Supplementary Table 1. Frailty Index creation. [file 12877_2022_3355_MOESM1_ESM.docx]

| Supplementary Table 1: Frailty Index creation | | | | |
| --- | --- | --- | --- | --- |
| Item | Type | Scoring: | Prevalence (%) |  |
| General Health  “baseline_generalhealth_fi” | Self rated | Excellent, Very good, good (1,2,3) = 0  Fair/Poor (4,5) = 1 | 0 = 82.0%  1 = 17.0%  Missing = 1% | 1 |
| Myocardial Infarction  'mi_fi' | Comorbidity | Yes = 1  No =0 | 0 = 78.6%  1 = 20.8%  Missing = 0% | 2 |
| Hypertension  'htn_fi' | Comorbidity | Yes = 1  No =0 | 0=49.6%  1=49.4%  Missing =0% | 3 |
| Diabetes  'dm_fi' | Comorbidity | Yes = 1  No =0 | 0=87.4  1=12.1  Missing =0 | 4 |
| Stroke  'stroke_fi' | Comorbidity | Yes = 1  No =0 | 0=90.5  1=8.9  Missing =0 | 5 |
| Cancer  'cancer_fi' | Comorbidity | Yes = 1  No =0 | 0=75.9  1=23.6  Missing=0.0 | 6 |
| COPD  'copd_fi' | Comorbidity | Yes = 1  No =0 | 0=85.7  1=13.7  Missing=0.6 | 7 |
| Read Newspaper  'baseline_readingpaper (problemvisionhearing)_fi' | Sensory | No or Little Difficulty (1,2) = 0  Some or Great Deal Difficulty (3,4) = 1 | 0=96.6  1=2.8  Missing=5.9 | 8 |
| Read Signs  'baseline_readingsigns (problemvisionhearing)_fi' | Sensory | No or Little Difficulty (1,2) = 0  Some or Great Deal Difficulty (3,4) = 1 | 0=91.1  1=8.2  Missing=0.7 | 9 |
| Hear Converstations  ' baseline_hearingconversation (problemvisionhearing)_fi' | Sensory | No or Little Difficulty (1,2) = 0  Some or Great Deal Difficulty (3,4) = 1 | 0=97.1  1=2.5  Missing=0.4 | 10 |
| Hear Noisy Room  'baseline_hearinginnoisyroom (problemvisionhearing)_fi' | Sensory | No or Little Difficulty (1,2) = 0  Some or Great Deal Difficulty (3,4) = 1 | 0=72.8  1=26.5  Missing=0.6 | 11 |
| Urinary Incontinence  'baseline_urineleak_fi' |  | Less than once a week (0,1,2) = 0  At least once a day (3,4,5) = 1 | 0=88.9  1=10.5  Missing=0.6 | 12 |
| Fall by accident  'baseline_fallenbyaccident_fi' | Function | No (2) = 0  Yes (1) = 1 | 1=64.3  0=34.6  Missing=1.0 | 13 |
| Weight loss  'baseline_weightloss_fi' |  | Loss >3kg (0) = 1  Anything less than 3kg (1,2,3) = 0 | 0=97.1  1=2.4  Missing=0.4 | 14 |
| Mobility  'baseline_mobility.1_fi' |  | Immobile or requires 1 person (0,1,2) = 1  Independent with or w/o aid (3) = 0 | 0=96.8  1=2.7  Missing=0.5 | 15 |
| Feed  'baseline_kitchen-kfeed_fi' | Function | Not at all, with help or alone with difficulty (1,2,3) = 1  Alone easily (4) = 0 | 0=98.4  1=1.2  Missing=0.3 | 16 |
| Make hot drink  baseline_kitchen-khotdrink_fi' | Function | Not at all, with help or alone with difficulty (1,2,3) = 1  Alone easily (4) = 0 | 0=95.6  1=4.0  Missing=0.3 | 17 |
| Wash up in kitchen  'baseline_kitchen-kwashingup_fi' | Function | Not at all, with help or alone with difficulty (1,2,3) = 1  Alone easily (4) = 0 | 0=94.3  1=5.3  Missing=0.4 | 18 |
| Finances  'baseline_domestic-dmoney_fi' | Function | Not at all, with help or alone with difficulty (1,2,3) = 1  Alone easily (4) = 0 | 0=93.5  1=5.9  Missing=0.6 | 19 |
| Laundry  'baseline_domestic-dsmallclothes_fi' | Function | Not at all, with help or alone with difficulty (1,2,3) = 1  Alone easily (4) = 0 | 0=89.5  1=9.8  Missing=0.6 | 20 |
| Shopping  'baseline_domestic-dshopping_fi' | Function | Not at all, with help or alone with difficulty (1,2,3) = 1  Alone easily (4) = 0 | 0=82.1  1=17.4  Missing=0.5 | 21 |
| Social activities  'baseline_leisure-lsocial_fi' | Function | Not at all, with help or alone with difficulty (1,2,3) = 1  Alone easily (4) = 0 | 0=87.4  1=11.8  Missing0.8 | 22 |
| Garden  'baseline_leisure-lgarden_fi' | Function | Not at all, with help or alone with difficulty (1,2,3) = 1  Alone easily (4) = 0 | 0=54.2  1=38.2  Missing=7.6 | 23 |
| Polypharmacy  'poly_fi' |  | > 5 Meds = 1  <5 Meds = 0 | 0=62.9  1=33.4  Missing=3.6 | 24 |
| Past Delirium  'baseline_delirium_fi' |  | No = 0  Yes = 1 | 0=87.3  1=10.7  Missing=2.0 | 25 |
| Immediate Racall  'immediate_fi' | Cognitive | >4 words = 0  <4 words = 1  *25^th^ percentile | 0=64.4  1=35.6 | 26 |
| Delayed Recall  'delayed_fi' | Cognitive | >2 words = 0  <2 words = 1  *25^th^ percentile | 0=66.2  1=33.8 | 27 |
| Groom  'groom_fi' | Function | Independent (5) = 0  Needs help (0) = 1  *I believe as dictionary is different | 0=96.8  1=1.6  Missing=1.6 | 28 |
| Stairs  'stairs_fi' | Function | Independent (10,15) = 0  Unable to needs help (0,5) = 1  *I believe as dictionary is different | 0=93.6  1=5.8  Missing=0.6 | 29 |
| Transfer  'transfer_fi' | Function | Independent (15) = 0  Any help or unable (0, 5,10) = 1  *I believe as dictionary is different | 0=93.6  1=5.8  Missing=0.4 | 30 |
| Bath  'bath_fi' | Function | Independent, unsupervised (0) = 0  Dependent (5) = 1 | 0=95.8  1=3.7  Missing=0.5 | 31 |
| Dress  'dress_fi' | Function | 10 = 0  0,5 = 1 | 0=96.0  1=3.6  Missing=0.5 | 32 |
| Toilet  'toilet_fi' | Function | 10 = 0  0,5 = 1 | 0=98.0  1=1.6  Missing=0.4 | 33 |
| Verbal fluency (animals)  'animals_fluency_fi' | Cognitive | >10 words = 0  <10 words = 1 | 0=86.9  1=8.8  Missing=4.3 | 34 |
| Verbal fluency (letter words)  'letters_fluency_fi' | Cognitive | >10 words = 0  <10 words = 1 | 0=79.8  1=16.1  Missing=4.2 | 35 |


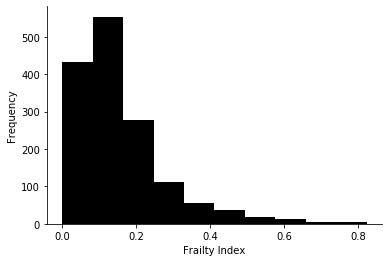


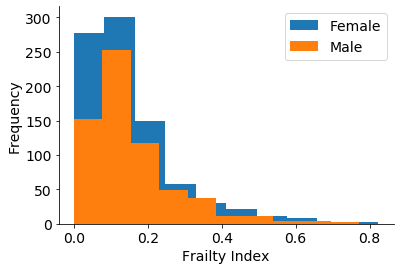


Cross Sectional Slope = 3.3%
