# Supplementary material for: Life-space, frailty, and health-related quality of life
Source: BMC Geriatr. 2022 Aug 6;22:646. doi: 10.1186/s12877-022-03355-2 (PMC9356461; doi:10.1186/s12877-022-03355-2)
Supplement: Supplementary file 2 — Additional file 2: Supplementary Table 2. Variables associated with life-space (n=1,510). [file 12877_2022_3355_MOESM2_ESM.docx]

| Supplementary Table 2: Variables associated with life-space (n=1,510) | | | | | | | | | |
| --- | --- | --- | --- | --- | --- | --- | --- | --- | --- |
| Life-space | Unadjusted | | | |  | Multivariate Linear Regression of 1,493 individuals | | | |
|  | Coef. | [95% Conf. Interval] | | *P* |  | Coef. | [95% Conf. Interval] | | *P* |
|  |  |  |  |  |  |  |  |  |  |
| Age | -5.3 | -6.0 | -4.6 | <0.001 |  | -2.5 | -3.2 | -1.7 | <0.001 |
|  |  |  |  |  |  |  |  |  |  |
| Women (cf.) men | -1.4 | -3.0 | 0.3 | 0.102 |  | -2.0 | -3.4 | -0.5 | 0.007 |
|  |  |  |  |  |  |  |  |  |  |
| Frailty Index | -8.2 | -8.9 | -7.4 | <0.001 |  | -6.6 | -7.4 | -5.7 | <0.001 |
|  |  |  |  |  |  |  |  |  |  |
| IMD | -1.4 | -2.2 | -0.5 | 0.001 |  | 0.02 | -0.7 | 0.8 | 0.962 |
|  |  |  |  |  |  |  |  |  |  |
| Qualification |  |  |  |  |  |  |  |  |  |
| GCSE, AS or A levels | [ref] |  |  | <0.001 |  | [ref] |  |  | 0.027 |
| HNC/HND or Diploma | -0.4 | -3.8 | 3.0 |  |  | -1.0 | -4.0 | 2.1 |  |
| Bachelor's or Postgraduate | 3.0 | 0.5 | 5.4 |  |  | 0.1 | -2.2 | 2.5 |  |
| No qualifications | -8.9 | -12.0 | -5.9 |  |  | -3.5 | -6.3 | -0.7 |  |
|  |  |  |  |  |  |  |  |  |  |
| Occupational skill |  |  |  |  |  |  |  |  |  |
| Level 1 | [ref] |  |  | <0.001 |  |  |  |  | 0.704 |
| Level 2 | 4.8 | 0.9 | 8.7 |  |  | 1.6 | -1.8 | 5.0 |  |
| Level 3 | 7.3 | 3.4 | 11.2 |  |  | 1.0 | -2.5 | 4.5 |  |
| Level 4 | 9.3 | 5.8 | 12.8 |  |  | 0.5 | -2.8 | 3.8 |  |
| Cf. = compared with.  IMD = Index of Multiple Deprivation | |  |  |  |  |  |  |  |  |
