# Supplementary material for: Life-space, frailty, and health-related quality of life
Source: BMC Geriatr. 2022 Aug 6;22:646. doi: 10.1186/s12877-022-03355-2 (PMC9356461; doi:10.1186/s12877-022-03355-2)
Supplement: Supplementary file 3 — Additional file 3: Supplementary Table 3. Variables associated with HRQoL (with frailty in levels) [file 12877_2022_3355_MOESM3_ESM.docx]

| Supplementary Table 3: Variables associated with HRQoL (with frailty in levels) | | | | |
| --- | --- | --- | --- | --- |
|  | EQ-5D Index |  |  |  |
|  | Multivariate Linear Regression of 943 individuals | | | |
|  | Coef. | [95% conf. interval] | | *P* |
| Age | 0.01 | 0.0002 | 0.02 | 0.045 |
| Female (cf.) Men | -0.02 | -0.04 | -0.003 | 0.020 |
| Frailty |  |  |  |  |
| Low | [ref] |  |  | <0.001 |
| Medium | -0.04 | -0.06 | -0.02 |  |
| High | -0.1 | -0.1 | -0.1 |  |
| Life-space | 0.01 | -0.01 | 0.03 | 0.319 |
| Frailty#Life-space |  |  |  |  |
| Low | [ref] |  |  | <0.001 |
| Medium | 0.02 | -0.002 | 0.04 |  |
| High | 0.06 | 0.03 | 0.08 |  |
| Loneliness | -0.01 | -0.04 | 0.01 | 0.304 |
| Lives alone | -0.01 | -0.03 | 0.01 | 0.544 |
| Care package |  |  |  |  |
| None | [ref] |  |  | <0.001 |
| Weekly | -0.9 | -0.2 | -0.01 |  |
| Daily/multiple times daily | -0.1 | -0.2 | -0.1 |  |
| Alcohol intake |  |  |  |  |
| Daily | [ref] |  |  | 0.674 |
| Weekly | -0.001 | -0.02 | 0.02 |  |
| Monthly or less | -0.01 | -0.03 | 0.01 |  |
| Cf. = compared with. |  |  |  |  |
