# Supplementary figures and images for: Life-space, frailty, and health-related quality of life
Source: BMC Geriatr. 2022 Aug 6;22:646. doi: 10.1186/s12877-022-03355-2 (PMC9356461; doi:10.1186/s12877-022-03355-2)

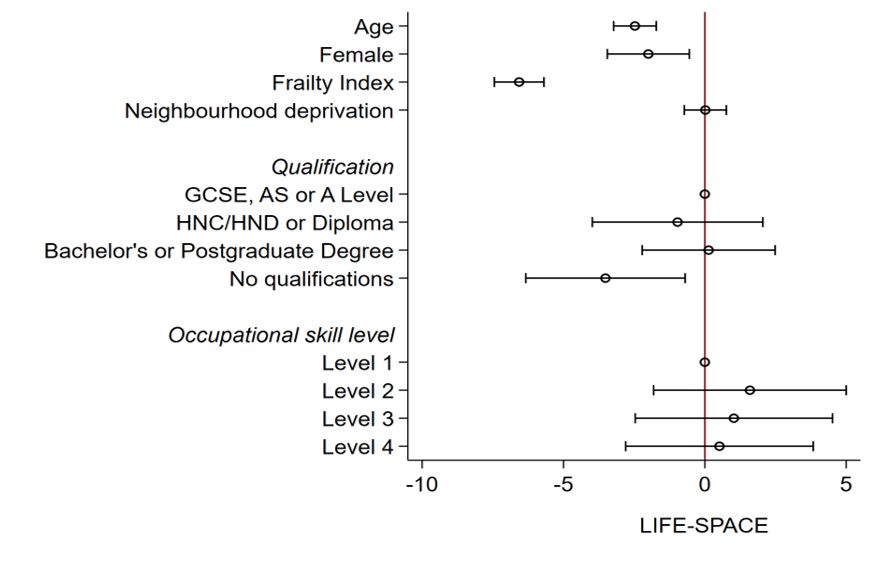


Supplementary Figure 1: Variables associated with life-space.

Supplement: Supplementary file 4 — Additional file 4: Supplementary Figure 1. Variables associated with life-space. [file 12877_2022_3355_MOESM4_ESM.docx]
